# Thrombin generation and platelet activation in cytoreductive surgery combined with hyperthermic intraperitoneal chemotherapy - A prospective cohort study

**DOI:** 10.1371/journal.pone.0193657

**Published:** 2018-06-21

**Authors:** Sven Van Poucke, Dana Huskens, Kurt Van der Speeten, Mark Roest, Bart Lauwereins, Ming-Hua Zheng, Seppe Dehaene, Joris Penders, Abraham Marcus, Marcus Lancé

**Affiliations:** 1 Department of Anesthesiology, Intensive Care, Emergency Medicine and Pain Therapy, ZOL, Genk, Belgium; 2 Synapse Research Institute, Maastricht, The Netherlands; 3 Department of Surgery, ZOL, Genk, Belgium; 4 Department of Hepatology, the First Affiliated Hospital of Wenzhou Medical University, Wenzhou, China; 5 Central Diagnostic Laboratory,ZOL, Genk, Belgium; 6 Department of Anesthesiology, ICU and Perioperative Medicine,HMC, Doha,Qatar; 7 Department of Anesthesiology & Pain Treatment, Maastricht University Medical Centre, Maastricht, The Netherlands; Public Library of Science, UNITED KINGDOM

## Abstract

**Background and objectives:**

Cytoreductive surgery (CRS) with hyperthermic intraperitoneal peroperative chemotherapy (HIPEC), indicated for patients with peritoneal metastases from digestive or gynecological malignancies alike, demonstrates a considerable impact on hemostatic metabolism, both on platelet and on coagulation level. The potential hemostatic interference in CRS and HIPEC is phase dependent. The hypothesis of this prospective cohort study is that the procedure exposed an increased thrombotic risk, resulting in a faster and increased thrombin generation and hyper platelet function.

**Methods:**

This study explores the combined use of ROTEM (rotational thromboelastometry), PACT (platelet activation test) and CAT (thrombin generation test) assays during CRS and HIPEC with a follow-up of 7 days postoperative in 27 patients with confirmed histological diagnosis of peritoneal disease.

**Results:**

Platelet reactivity (relative to before incision values) to CRP (collagen-related peptide) (p value 0.02) and TRAP (thrombin receptor activator peptide) (p value 0.048) seems to be slightly reduced during CRS and HIPEC with regard to αIIbβ3 activation, while P-selectin expression is not affected. During surgery, CAT demonstrates that, the LT (lagtime) (p value 0.0003) and TTP (time-to-thrombin peak) values (p value 0.002) decrease while and the TP (thrombin peak) (p value 0.004) and ETP (endogenous thrombin potential) (p value 0.02) increase. Subsequently, after surgery, the LT and TTP increase and ETP and TP decrease in time. ROTEM EXTEM (extrinsic) MCF (maximum clot firmness) (p value 0.005), INTEM (intrinsic) MCF (p value 0.003) and FIBTEM (fibrinogen) MCF (p value <0.001) decreased during CRS. At day 7 INTEM and FIBTEM MCF values (p values of 0.004 and <0.001) were significantly higher than before surgery. No considerable changes in platelet count and hemoglobin concentration and absence of leukopenia are noticed.

**Conclusion:**

This approach detects changes in coagulation much earlier than noticed by standard coagulation tests.

## Introduction

Cytoreductive surgery (CRS) with hyperthermic intraperitoneal peroperative chemotherapy (HIPEC) is a therapeutic modality for patients with abdominal malignancies and peritoneal dissemination (quantified by the peritoneal cancer index (PCI) [1,2]. CRS and HIPEC were introduced in 2001 in our facility. Since then, over 620 CRS/HIPEC procedures have been accomplished. The procedure consists of surgical removal of macroscopic tumor tissue, combined with intraperitoneal/IV, heated chemotherapy perfusate administered during surgery in the abdomen with the aim of annihilating the microscopic residual tumor volume [2]. The thermal enhancement and pharmacokinetics of chemotherapeutic agents have been extensively described [3–6]. CRS and HIPEC potentially exert a substantial impact on the patients’ respiratory, cardiovascular, renal, and metabolic status [7–10]. Additionally, the procedure has been depicted as a high risk with significant levels of comorbidity (22–39%), mortality (5%) and prolonged hospital stays [11]. Although cytoreductive surgery (CRS) and hyperthermic intraperitoneal preoperative chemotherapy (HIPEC) have established its therapeutic role in selected patient populations with peritoneal carcinomatosis (PC), multiple factors can disrupt the patient metabolism during and after surgery [12–14].

Complications, as assessed by the disease burden (PCI) depend on the experience of the surgical team [2, 15, 16]. In general, the majority of complications are situated in three groups: one third of the patients develop digestive fistulas, one third pulmonary complications, and one third hematological complications [17]. The multifactorial impact of surgery (open abdomen or Coliseum technique), hyperthermia and chemotherapy on hemostatic physiology of oncologic patients remains to be quantitatively elucidated, although both a potential bleeding risk as a potential thrombotic risk have been related with CRS-HIPEC [17]. The purpose of this study was to quantitatively assess the impact of CRS and HIPEC, on various components of hemostasis. Routine laboratory assays such as activated clotting time, activated partial thromboplastin time, prothrombin time, or platelet count might, as demonstrated previously, insufficiently provide specificity and/or sensitivity to assess coagulation and acquired platelet function disorders. Therefore, additionally thrombin generation (TG) was analyzed by the calibrated automated thrombogram assay (CAT) [18–20]. Also, platelet function was quantitatively assessed by the PAC-t-UB assay and rotational thromboelastometry (ROTEM) was used to elucidate the contribution of platelets, intrinsic and extrinsic coagulation pathways in peri-operative bleeding [21–24]. The hypothesis of this study was that the procedure exposed an increased thrombotic risk, resulting in a faster and increased thrombin generation and hyper platelet function.

## Methods & materials

### Study design and patients

This prospective observational pilot study, recruited patients scheduled for CRS-HIPEC between April 2015 and July 2016, finally included 27 patients from the Ziekenhuis Oost-Limburg, Genk, Belgium. One patient refused enrollment. The study was approved by the local medical ethics committee (Comité Medische Ethiek ZOL Genk Belgium) (B371201524199), written informed consent was obtained from the enrolled patients. Eligibility criteria included: a confirmed histological diagnosis of peritoneal disease (e.g., mesothelioma; pseudomyxoma peritonei; colorectal, ovarian, or gastric peritoneal carcinomatosis of colorectal, ovarian, or gastric cancer origin; or abdominal sarcomatosis); age <80 years; a cardiac, renal, hepatic, and bone marrow function compatible with surgery; and informed written consent to participate in the study. Exclusion criteria were: inherited coagulation abnormalities, active systemic infections, interstitial lung disease, serious cardiac dysrhythmia or condition, New York Heart Association classification of III or IV, uncontrolled hypertension (diastolic blood pressure constantly >100 mm Hg, systolic blood pressure constantly > 180 mm Hg). Inadequate bone marrow function at the beginning of the trial, defined as platelet count less than <150 GPT/L (gigapartikel/liter) or neutrophil granulocyte count less than <1.5 GPT/L. Inadequate renal function at the beginning of the trial, defined as GFR (glomerular filtration rate) less than <60 ml/min, inadequate liver function at the beginning of the trial, defined as bilirubin >1.5 times ULN (upper limit of normal), active hepatitis B or C infection, female patients who are pregnant or breast feeding and participation in another therapeutic clinical trial.

Data were collected from inclusion, the day before surgery and consisted of the patient characteristics, anesthesia procedure, peri-operative fluid administration, transfusion and coagulation management and body temperature. Laboratory values and blood gas analysis were recorded before incision, before start chemotherapy, at 30 (and if relevant at 60–90) minutes after the start of chemotherapy depending on the cycle length, at the 1^st^ and 7^th^ day postoperative. At the end of the surgical procedure, all patients were admitted to a postoperative intensive care unit for at least 24 h. Postoperative data included pain management, surgical drainage, immediate complications were additionally recorded. Primary outcome measure was per-operative blood loss, secondary outcome measures were parameters as further described by standard hematological analyses, thrombin generation assay, platelet activation test, rotational thromboelastometry.

### Surgical technique and anesthesia

On arrival at the operating theater, standard monitoring (i.e. electrocardiography, noninvasive blood pressure measurement, plethysmographic oximetry, rectal temperature) was initiated. Hereafter, a venous line (Vasofix® Safety, 14G or 16G, B.Braun Melsungen, Germany) was placed in the forearm and an arterial line (Radial Artery Catheterization set, 20 G, Arrow International, Reading, MA, USA) was positioned in the radial artery using subcutaneous local anesthesia (1 ml of lidocaine 1% solution). An epidural catheter was introduced at the levels of L1-L3 before the induction of anesthesia unless otherwise contra-indicated. Anesthesia was induced intravenously, with remifentanyl 0.25–1 mcg/kg/min and propofol TCI (target-controlled infusion) 4 to 6 ng/mL given intravenously, and intubation was facilitated with rocuronium 0.5 mg kg−1. All patients received 2g cephazolin and 1500 mg metronidazol at the end of the induction. Ventilation was set to achieve normal values of blood gases; anesthesia was maintained with air/oxygen mixture, maintenance shot doses of relaxant and supplemented with fentanyl 50–100 μg shots, or epidural marcaine 0.25% 10–15 ml bolus and 5 ml shot doses throughout the surgery.

The generic surgical approach involved peritonectomy procedures and visceral resections called CRS as described by Sugarbaker (1995). Peritoneal disease burden was assessed using the peritoneal cancer index (PCI), which scores 13 intra-abdominal sites on a scale of 0 (no disease) to 3 (lesion size > 5 cm), thus giving a range of possible scores from 0 to 39. The same team performed the surgical procedure of all included patients. Before connection to the patient, the circuit was filled with dextrose 5% (2 L/m2 body surface area) and warmed to 37°C.

Normovolemia was maintained by ensuring appropriate intravenous fluid and blood replacement for insensible fluid and blood losses that may be gradual and may accumulate over the long duration of surgery. End points such as the maintenance of blood pressure within 20% of the patient’s baseline and urine output > 0.5 mL/kg/hour were targeted. Blood product replacement was guided by clinical estimation of blood loss and targeted hemoglobin levels of 8–10 mg/dL. Fresh frozen plasma was given in an effort to stabilize prothrombin time (PT)/international normalized ratio (INR) below 1.2. Base excess and pH measured intraoperatively also gave indications of the adequacy of tissue perfusion. Normothermia was sustained by forced air warming or underbody thermal blanket, bearing in mind that the subsequent HIPEC tended to increase patient’s body temperature.

### Chemotherapeutic agents

The intraperitoneal temperature was maintained at 41.5–42.5°C during the perfusion. Different chemotherapeutic agents were used for bidirectional chemotherapy, depending on the tumor’s histological characteristics. Colon and appendix cancer: *oxaliplatinum* 460 mg/m^2^ intraperitoneal for 30 minutes plus a fast central IV bolus of *5-fluorouracil* 400 mg/m^2^ in 250ml NaCl 0.9% and a fast peripheral IV bolus of *leukovorin (folinic acid)* 20mg/m^2^ in 250ml NaCl 0.9%. Gastric, ovarian cancer and mesothelioma: *cisplatinum* 50 mg/m^2^ and *doxorubicin* 15 mg/m^2^ for 90 minutes intraperitoneal associated with IV *ifosfamide* 1300 mg/m^2^ over 90 minutes. Mesna (sodium-2mercaptoethane sulfonate) was used to protect against hemorrhagic cystitis.

### Blood collection and laboratory analyses

Blood samples were collected in vacuum tubes via the arterial line, using a VenoJect® Quick Fit luer adapter (XX-MN2000Q,Terumo Medical, Europe), at different time points: before incision (baseline), before chemotherapy, after 30, 60, 90 minutes of chemotherapy and on day 1,3 and 7. Following discarding 10 mL of blood at each time point, 4 mL whole blood was collected in a K2EDTA 7.2 mg BD Vacutainer® (Ref.: 368861, Becton,Dickinson & Company, Plymouth, UK), 4.5 mL whole blood in a sodium citrate 3.2% BD Vacutainer® (Ref.: 367714, Becton, Dickinson & Company, Plymouth, UK), and 3 mL whole blood in a hirudin 15 μg/mL vacutainer (Ref.: MP0600, Verum Diagnostica GmbH, Munich, Germany). Blood samples were directly transported to the laboratory and analyzed within 2–4 h after collection to allow for minimal necessary resting time. ROTEM, PACT and thrombin generation assays were performed as further described.

#### Standard hematological analyses

EDTA-anticoagulated blood was used for cytometric analysis using a whole blood counter Sysmex XE 2100® (Sysmex,Kobe, Japan) to obtain a whole blood count. Fibrinogen levels were determined with an ACL-9000 (Diamond Diagnostics, Holliston, MA) coagulation analyser, using a PT-fibrinogen high sensitivity reagent, which is a high-sensitivity calcium thromboplastin that allows the simultaneous determination of PT and fibrinogen levels. aPTT was measured using an ACL-9000 coagulation analyser and INR was calculated by the formula INR (PT patient/PT normal).

#### Thrombin generation assay

TG in plasma was measured with the calibrated automated thrombogram (CAT) assay as developed by Hemker and co-workers [18–20]. Briefly, 80 µl platelet poor plasma (PPP) was mixed with 20 µl of a mixture containing tissue factor (Dade-Behring) at a final concentration of 1 pM and phospholipid vesicles (f.c. 4 µM 20 mol% phosphatidylserine, 60 mol% phosphatidylcholine and 20 mol% phosphatidyl-ethanolamine, Avanti). To calibrator wells, 20 µl of calibrator (α_2_macroglobulin-thrombin complex, [19]) was added instead of TF and PL. After 10 minutes of incubation at 37°C, thrombin generation was initiated by the addition of 20 μl of the thrombin specific substrate, Z-Gly-Gly-Arg-7-amino-4-methylcoumarin (f.c. 416 µM, Bachem) and CaCl2 (f.c. 16.7 mM). Fluorescence was measured with a Fluoroscan Ascent reader (Thermo Labsystems) and data were analyzed with dedicated software (Thrombinoscope, Stago) [20]. Thrombin generation was expressed based on endogenous thrombin potential (ETP); lagtime (LT); thrombin peak (TP), time-to-thrombin peak (TTP).

#### Platelet activation test

Platelet activation was quantitatively assessed on a randomly selected subgroup of 10 patients (not treated with acetylsalicylic acid) in un-processed blood by the PACT (Platelet activation test, adjusted from Roest 2013). In the original paper, Roest performs the tests at room temperature while in this manuscript, the PACT test is performed at 37°C which requires higher agonist doses.

The PACT is based on platelet activation induced by addition of a specific agonist to whole blood and gives specific insight in the granule release capacity and in the aggregation potential of platelets. The test contains three agonists to activate the platelets: (1) the protease activated receptor (PAR-1) agonist thrombin receptor activator peptide (TRAP f.c. 30 µM, SFLLRN, H-2936; Bachem, Germany), (2) the glycoprotein VI (GPVI) agonist collagen-related peptide (CRP f.c. 5 µg/ml, Professor Farndale, university of Cambridge, UK), and (3) the P_2_Y12 agonist ADP (f.c. 30 µM, 01897, Sigma-Aldrich, Zwijndrecht, the Netherlands) in HEPES-buffered saline (HBS, 10 mmol/L HEPES, 150 mmol/L NaCl, 1 mmol/L MgSO4, 5 mmol/L KCL, pH 7.4). The reaction mixtures also contain three antibodies directed against GPIb (APC-conjugated CD42b, BD Bioscience), activated αIIbβ3 (FITC-conjugated PAC-1) and P-selectin (PE-conjugated CD62P) purchased from BD Pharmingen (Franklin Lakes, USA). Whole blood was heated at 37°C for 10 min and the tests were performed at 37°C. Whole blood was diluted 1:4 in HEPES-buffered saline and 5 µl of this diluted blood was added to each reaction mixture. Reactions were stopped by adding 250 µl fixation solution (137 mmol/L NaCl, 2.7 mmol/L KCl, 1.12 mmol/L NaH_2_PO_4_, 1.15 mmol/L KH_2_PO_4_, 10.2 mmol/L Na_2_HPO_4_, 4 mmol/L EDTA, 0.5% formaldehyde) after exactly 20 min of incubation at 37°C. Flow cytometry was used to distinguish between platelets and other cells on forward and sideward scatter pattern and by gating on the CD42b positive cells. Fluorescent intensity in the FITC gate and PE gate was selected to determine activated αIIbβ3 and P-selectin density, respectively, and results are expressed as median fluorescent intensity (MFI).

#### Rotational thromboelastometry

Thrombus formation was measured by the viscoelastic test: ROTEM (Tem International GmbH c/o Dutch Affiliate, Tilburg, The Netherlands). Standard assays were used according to the manufacturer’s recommendations: EXTEM (ref.: 503–05, Tem International GmbH c/o Dutch Affiliate, Tilburg, The Netherlands), FIBTEM (ref.: 503–06, Tem International GmbH c/o Dutch Affiliate, Tilburg, The Netherlands), and HEPTEM (ref.: 503–09, Tem International GmbH c/o Dutch Affiliate, Tilburg, The Netherlands). All samples were measured within 1 h after blood collection. Furthermore, by means of EXTEM and FIBTEM results, the contribution of platelet count to the thrombus formation was calculated as the PLTEM parameter [22].

All laboratory protocols were uploaded to protocols.io.

The following DOI’s are linked per test:

Standard hematological analyses HIPEC:

https://dx.doi.org/10.17504/protocols.io.ki6cuhe

PACT (Platelet Activation Test): https://dx.doi.org/10.17504/protocols.io.ki8cuhw

ROTEM (Rotational Thromboelastometry): https://dx.doi.org/10.17504/protocols.io.ki9cuh6

Thrombin generation assay (CAT):

https://dx.doi.org/10.17504/protocols.io.ki7cuhn

### Statistical analysis

Statistical analyses were performed using RapidMiner (7.2, Boston, MA) or GraphPad Prism 5.00. Graphical data are displayed as median with interquartile ranges and the Wilcoxon matched-pairs signed-ranks test for nonparametric variables was used to determine statistical significance (P<0.05). The statistical analysis is based on the same approach as published by this group in several papers.

## Results

### Study population

The patients included in this study were aged on average 63 years (range 36–76) with equal gender distribution. Seven patients more were included additional to the 20 patients initially planned in order compensate for potential data or sample losses which was not the case. Patient height and weight were respectively 170±9 cm and 72±12 kg (mean±SD) with ASA classification 2±1 (mean±SD). Five % of the patients were treated for diabetes mellitus, 40% for arterial hypertension. A quarter of the patients used nicotine the week before surgery, whereas 55% described a positive smoking history. About a third (35%) of the patients consumed alcohol in the week before the HIPEC procedure. One out of four patients were treated with acetylsalicylic acid. Every patient received a low-molecular-weight heparin (prophylactic dose) the evening before surgery. Postoperative, low-molecular-weight heparin was reinitiated as soon as the surgical drainage stopped (median: day 2 postoperative). The assessment of carcinomatosis extension among the patients revealed a mean PCI score of 15 (range 3–29) with a mean prior score of 1 (range 1–3). The origin of carcinomatosis consisted of primarily digestive cancer followed by peritoneal carcinomatosis from the ovarian. The median hospital stay was respectively 23 days (range 5–87).

### Blood loss

Blood loss during surgery was 123 ± 88 ml, mean operation theatre time was 493 (range 293–800) minutes. Re-operation to treat bleeding complication within the study period was required in 2 patients. Transfusion during surgery was administered in 15%, 10 and 0% of the patients, respectively for packed cells, fresh frozen plasma and platelets. The total average amount of fluid therapy during surgery was 4325 ± 2015 ml. Postoperative blood transfusion during ICU stay was required in 22.2% of the patients, FFP in 3.7% and platelets in 7.4% of the patients. Results of all measurements are illustrated in Table 1.

**Table 1:** Effect of HIPEC on laboratory tests, blood gas analysis and ROTEM.

	Before incision		Before Chemo		30 min chemo		Day 1		Day 3		Day7	
	median	IQR	median	IQR	median	IQR	median	IQR	median	IQR	median	IQR
**Laboratory tests:**												
Hemoglobin (g/dl)	10.9	10.0–12.6	10.8	9.5–12.4	11.1	9.3–12.2	12.1	10.1–12.9	10.3	9.1–11.9	11.1	9.9–12.4
Platelet count (*10^9^/l)	205	165–267	196	156–238	198	185–280	191	142–201	164	85–193	238	123–306
WBC (/mm^3^)	7.0	3.9–8.3	8.5	6.4–11.7	9.8	8.0–10.9	10.5	8.8–12.5	6.4	5.4–8.5	8.0	7.0–9.7
Ca2+ (mmol/l)	4.43	4.35–4.51	4.33	4.14–4.50	4.25	4.20–4.40	4.43	4.38–4.58	4.70	4.65–4.80	4.61	4.41–4.79
Glucose (mg/dl)	109	93–118	135	117–138	360	257–414	144	129–164	127	107–150	146	132–228
**Blood gas analysis:**												
pH	7.35	7.34–7.39	7.35	7.33–7.37	7.34	7.32–7.35	7.39	7.38–7.41	7.41	7.40–7.43	7.42	7.40–7.45
Lactate (mmol/l)	0.7	0.7–1.0	0.7	0.6–1.0	2.9	1.7–4.1	1.3	1.1–1.9	1.0	0.7–1.3	1.3	1.2–2.2
**Hemostasis:**												
aPTT (sec)	31.6	29.3–33.1	31.4	29.6–33.2	31.1	29.8–32.5	35.7	32.0–37.8	33.9	31.8–36.8	31.8	29.8–34.9
PT (%)	80	69–85	76	71–84	75	71–78	64	59–74	81	76–86	76	66–84
INR	1.15	1.10–1.27	1.18	1.11–1.24	1.19	1.15–1.23	1.31	1.21–1.40	1.13	1.10–1.20	1.18	1.11–1.31
Fibrinogen (g/dl)	366	304–411	306	236–358	275	231–335	392	346–421	528	462–574	627	582–731
**ROTEM:**												
EXTEM A5 (mm)	47	41–52	43	37–46	42	38–48	45	41–51	48	41–53	51	45–60
EXTEM A30 (mm)	66	61–71	63	60–65	62	59–67	65	60–67	65	59–70	71	64–77
EXTEM alpha (°)	78	70–80	73	67–76	69	65–78	79	74–80	80	78–82	80	78–81
EXTEM CFT (s)	88	68–112	104	81–135	108	80–130	84	73–103	72	64–90	72	52–90
EXTEM CT (s)	48	45–54	49	44–67	49	43–56	51	48–53	48	43–54	57	51–67
EXTEM MCF (mm)	66	61–70	63	60–65	62	59–67	65	61–67	66	59–70	68	61–74
EXTEM ML (%)	4	3–4	4	1–6	2	1–3	7	4–10	8	6–13	4	2–7
FIBTEM A5 (mm)	14	11–19	10	8–16	9	7–15	13	12–18	19	17–22	21	19–27
FIBTEM A30 (mm)	17	14–24	13	10–20	12	9–19	17	15–23	24	21–28	25	23–33
FIBTEM alpha (°)	77	71–80	73	60–79	76	70–79	76	72–81	79	79–82	80	79–81
FIBTEM CFT (s)	297	260–560	947	577–1311	832	491–1540	357	270–413	320	169–736	228	76–437
FIBTEM CT (s)	49	47–51	51	46–54	50	47–55	49	47–54	50	44–54	60	48–67
FIBTEM MCF (mm)	17	13–24	12	10–20	11	9–19	17	14–22	25	21–28	25	24–34
FIBTEM ML (%)	0	0–0	0	0–0	0	0–0	0	0–0	0	0–0	0	0–0
INTEM A5 (mm)	52	44–56	47	41–50	49	43–53	48	47–56	52	42–57	55	50–64
INTEM A30 (mm)	67	62–71	64	60–66	64	60–68	64	61–67	66	60–70	71	66–76
INTEM alpha (°)	79	74–80	76	72–78	77	74–79	77	76–80	79	78–80	80	79–81
INTEM CFT (s)	59	51–86	77	61–90	66	57–92	68	54–76	56	49–80	57	44–60
INTEM CT (s)	156	149–163	149	142–180	151	136–185	148	143–160	148	143–162	144	139–160
INTEM MCF (mm)	67	62–71	65	60–66	65	61–67	64	62–68	67	60–71	71	67–76
INTEM ML (%)	3	2–4	3	0–5	2	1–4	7	4–9	8	5–12	4	2–6

Data are presented as median and range. Significant changes compared to before incision values are underlined. IQR interquartile range; WBC white blood cells; aPTT activated partial thromboplastin time; PT prothrombin time; INR international normalized ratio; A5 (30) indicates amplitude at 5 (30) minutes; EXTEM external temogram; FIBTEM fibrinogen temogram; INTEM internal temogram; CFT clot formation time; CT clotting time; MCF maximum clot firmness; ML maximum lysis

### Cytoreductive surgery

The cytoreductive surgical phase was defined as the period from surgical incision until the start of chemotherapy administration. During this phase, we noticed that fibrinogen, Ca^2+^ concentration and the ROTEM maximum clot firmness of EXTEM, INTEM and FIBTEM significantly decreased (p values of 0.001, 0.010, 0.005, 0.003, <0.001, respectively). Moreover, a significant decrease was reported for EXTEM A5, A30 and alpha (p values of 0.004, 0.004 and 0.002, respectively) for INTEM A5, A30 and alpha (p values of 0.001, 0.009 and 0.001, respectively) and for FIBTEM A5, A30 and alpha (p values of <0.001, <0.001 and 0.001, respectively). On the contrary, white blood cell count and glycemia significantly increased (p values of 0.004 and 0.001, respectively). Moreover, CFT values of EXTEM, INTEM and FIBTEM significantly increased with p values of 0.002, 0.001 and 0.005, respectively. Compared to baseline (before incision), hemoglobin concentration, platelet count, prothrombin time, activated partial thromboplastin time, and both CT and ML of EXTEM, INTEM and FIBTEM did not demonstrate a significant change.

### Chemotherapy

The period of chemotherapy was defined as the phase starting immediately before chemotherapy administration until chemotherapy was stopped (the majority of the patients received 30 minutes of chemotherapy. This phase, demonstrated a significant increase in glucose and lactate concentration and FIBTEM alpha (p value of <0.001, < 0.001 and 0.017 respectively). A significant decrease was noticed for pH, prothrombin time, fibrinogen, EXTEM CT, FIBTEM A5, A30, alpha and CFT (p values of 0.004, 0.019, 0.019, 0.043, 0.045, 0.032 and 0.028, respectively). No significant changes were noticed for hemoglobin concentration, white blood cell count, platelet count, activated partial thromboplastin time and ROTEM EXTEM, INTEM and FIBTEM maximum clot firmness values. With only 4 patients that received chemotherapy for 60 minutes and only 3 patients, 90 minutes of chemotherapy, conclusions of chemotherapy were restricted to 30 minutes.

Compared to the moment before incision, CRS and 30 minutes chemotherapy resulted in a significant increase of WBC, glucose concentration, lactate, EXTEM CFT, INTEM CFT and FIBTEM CFT (p values of 0.001, <0.001, <0.001, < 0.001, 0.008 and 0.019, respectively) and a significant decrease in calcium concentration, pH, prothrombin time, fibrinogen (p values of 0.026, 0.011, 0.022 and <0.001, respectively). Moreover, a significant decrease was measured for EXTEM A5, A30, alpha, MCF and ML (p values of <0.001, 0.002, <0.001, 0.013 and 0.004, respectively), for INTEM A5, A30, alpha and MCF (p values of 0.011, 0.022, 0.009 and 0.017, respectively) and FIBTEM A5, A30 and MCF (p values of <0.001, <0.001 and < 0.001, respectively).

#### Day 1 postoperative

The phase defined as the period between incision and the first day postoperative provided values with significant increase for white blood cell count, glucose concentration, pH, lactate, activated partial thromboplastin time, INR, EXTEM ML, FIBTEM CFT and INTEM ML, (p values of <0.001, <0.001, 0.006, 0.003, 0.001, <0.001, 0.001, 0.003 and <0.001, respectively). A significant decrease was measured for platelet count, prothrombin time, EXTEM and INTEM maximum clot firmness values (p values of 0.001, <0.001, 0.004 and 0.004, respectively). Additionally, EXTEM A30, INTEM A30, FIBTEM ML were significantly decreased (p values of 0.002, 0.003 and 0.005, respectively).

Comparing values after 30 minutes of chemotherapy and 1 day postoperative demonstrated a significant increase of white blood cell count, calcium concentration, pH, aPTT, INR, fibrinogen, EXTEM A5, EXTEM alpha, EXTEM ML, INTEM alpha, INTEM ML, FIBTEM A5, FIBTEM A30 and FIBTEM MCF (p values of 0.048, 0.025, <0.001, 0.001, <0.001, <0.001, 0.027, <0.001, <0.001, 0.024, <0.001, <0.001, <0.001 and <0.001, respectively). A significant decrease was measured for platelet count, glucose concentration, lactate, prothrombin time, EXTEM CFT, FIBTEM CFT and FIBTEM ML (p values of 0.002, <0.001, 0.001, <0.001, 0.008, <0.001 and 0.003, respectively).

#### Day 3 postoperative

The phase between incision and the third day postoperative resulted in a significant increase of the calcium concentration, glucose concentration, pH, aPTT, fibrinogen, EXTEM alpha, EXTEM ML, INTEM alpha, INTEM ML, FIBTEM A5, FIBTEM A30, FIBTEM alpha and FIBTEM MCF (p values of <0.001, 0.010, <0.001, 0.014, <0.001, 0.001, <0.001, 0.037, 0.001, <0.001, <0.001, 0.002 and 0.001, respectively). A significant decrease on the other hand was observed for hemoglobin concentration, platelet count, EXTEM A30, INTEM A30 and FIBTEM ML (p values of 0.041, <0.001, 0.040, 0.041 and 0.005, respectively).

Comparing the results of day 3 postoperative with day 1 postoperative demonstrated a significant increase of calcium concentration, pH, prothrombin time, fibrinogen, EXTEM A5, EXTEM A30, EXTEM alpha, EXTEM MCF, EXTEM ML, INTEM alpha, FIBTEM A5, FIBTEM alpha, FIBTEM A30 and FIBTEM ML (p values of < 0.001, 0.015, 0.001, <0.001, 0.046, 0.030, 0.013, 0.032, 0.041, 0.007, <0.001, 0.001, <0.001 and 0.005, respectively) and a decrease of hemoglobin, platelet count, white blood cell count, glucose concentration, INR, EXTEM CFT, INTEM CFT (p values of 0.001, 0.004, <0.001, 0.009, <0.001, 0.004, and 0.038, respectively).

#### Day 7 postoperative

By comparing measurements on the 7^th^ day postoperative with before incision values, a significant increase was observed for white blood cell count, glucose, pH, lactate, fibrinogen, EXTEM A5, EXTEM A30, EXTEM alpha, EXTEM CT, INTEM A5, INTEM A30, INTEM alpha, INTEM MCF, FIBTEM A5, FIBTEM A30, FIBTEM alpha, FIBTEM CT and FIBTEM MCF (p values of 0.014, <0.001, 0.001, 0.001, 0.011, 0.016, 0.021, 0.037, 0.004, 0.003, 0.004, 0.004, 0.044, <0.001, 0.002, 0.018 and <0.001, respectively). A significant decrease was noticed for EXTEM CFT, INTEM CFT, FIBTEM CFT and FIBTEM ML with p values of 0.021, 0.012, 0.044 and 0.001, respectively.

### Platelet activation

**Fig 1:**
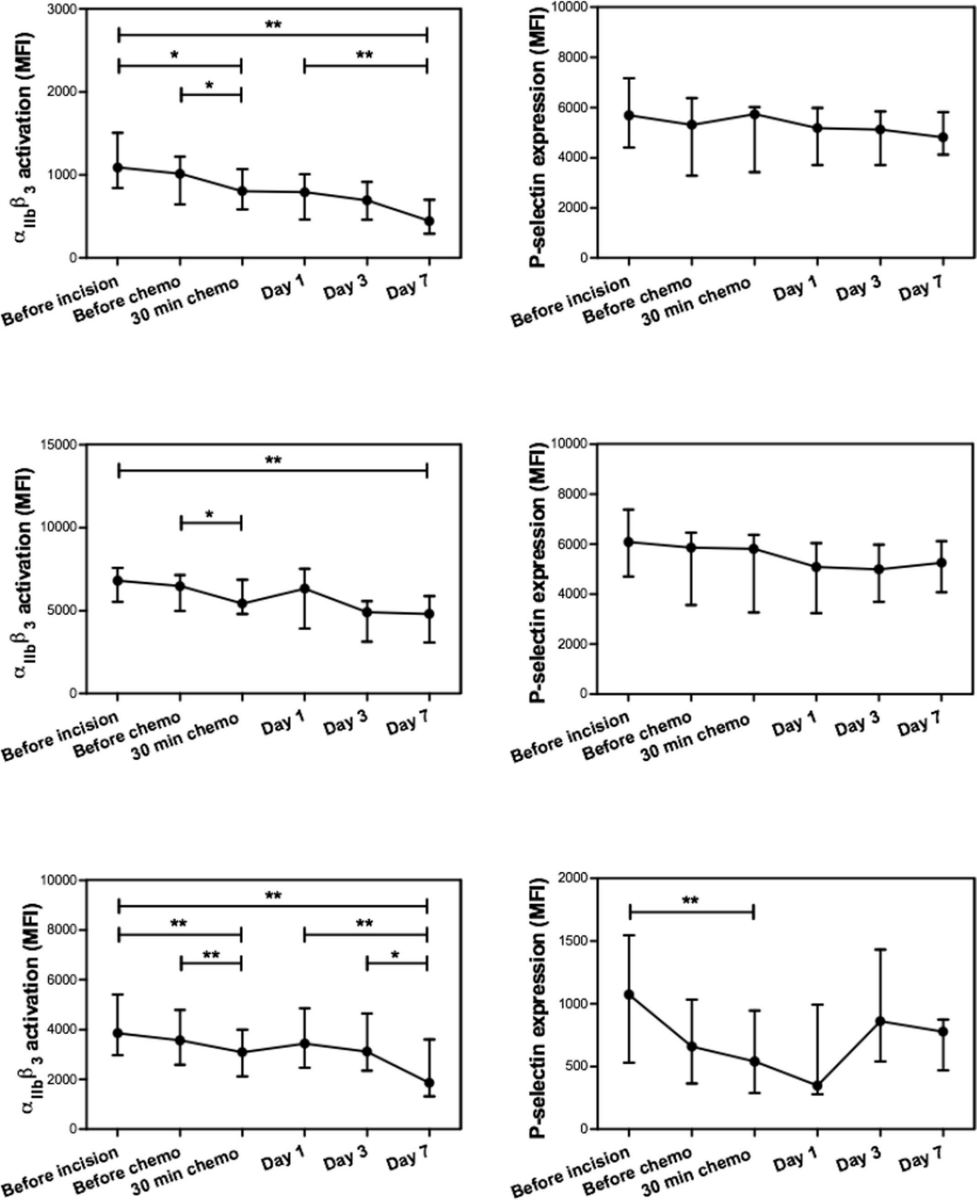
Effect of HIPEC on platelet function. TRAP, CRP and ADP were used to activate platelets via PAR-1 (panel A), GPVI (panel B) and P2Y_12_ receptor (panel C), respectively. Data of α_IIb_β_3_ activation (left) and P-selectin expression (right) are presented as medians with IQR. Significance was tested with the Wilcoxon matched-pairs signed rank test between two consecutive time points, between before incision and 30 minutes after chemotherapy, between day 1 and day 7 and between before incision and day 7. * P<0.05, ** P<0.01.

To study the effect of HIPEC on platelets function, we measured the granule release potential and the aggregation potential of the platelets by quantifying P-selectin expression and activation of the α_IIb_β_3_ receptor, respectively, after addition of PAR-1, glycoprotein VI (GPVI) (collagen) or P2Y_12_ receptor agonists ((n = 10), Fig 1 and Table 2). Chemotherapy resulted in a significant decrease of α_IIb_β_3_ receptor activation in response to TRAP, CRP and ADP (significant decrease 30 min after start chemo compared to before chemo with p values of 0.048, 0.02 and 0.01, respectively). During the days after surgery the α_IIb_β_3_ receptor activation in response to TRAP and ADP decreased even more, however, a large variation was observed between patients.

**Table 2:** Effect of HIPEC on platelet function.

	Before incision	Before Chemo		30 min chemo		Day 1		Day 3		Day 7	
	median	median	IQR	median	IQR	median	IQR	median	IQR	median	IQR
α_IIb_β_3_ activation											
TRAP	100	86	70–118	76	61–101	78	49–96	60	42–70	33	25–62
CRP	100	95	84–109	87	74–111	92	78–99	73	59–83	72	63–81
ADP	100	83	70–104	76	59–90	85	71–110	82	61–96	43	35–80
P-selectin expression											
TRAP	100	98	74–104	94	70–113	82	72–94	103	68–123	97	69–113
CRP	100	102	74–105	94	74–118	76	63–98	89	72–119	102	71–113
ADP	100	86	43–118	56	33–92	60	29–93	103	66–177	62	41–140

Data are presented as percentage of before incision. Significant changes compared to before incision values are underlined. TRAP thrombin receptor activator peptide; CRP collagen related peptide.

In contrast to the decrease in α_IIb_β_3_ receptor activation during chemotherapy, no difference in P-selectin expression was measured in response to the 3 agonists. Only a significant decrease in P-selectin expression in response to ADP was observed after 30 min of chemotherapy compared to before incision, however, here also a large variation was detected between patients. Results from unstimulated samples are not mentioned because no background activity was measured.

### Thrombin generation

**Fig 2:**
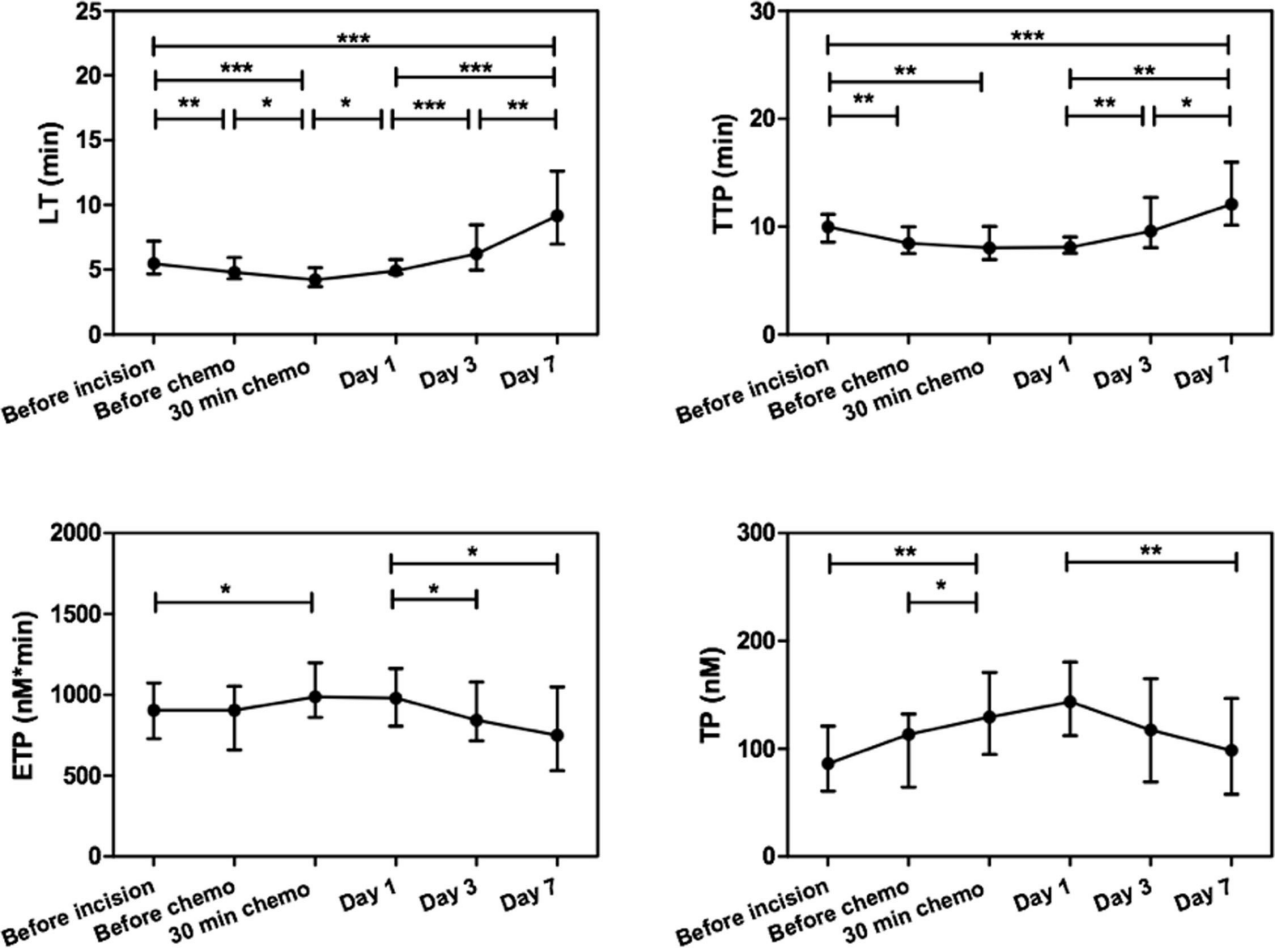
Effect of HIPEC on thrombin generation. TG was determined in PPP and, for each patient, ttPeak (TTP), thrombin peak (TP) and ETP values of the resulting thrombograms were calculated. Data are presented as median with IQR. Significance was tested with the Wilcoxon matched-pairs signed rank test between two consecutive time points, between before incision and 30 minutes after chemotherapy, between day 1 and day 7 and between before incision and day 7. * P<0.05, ** P<0.01, *** P<0.001.

To study the effect of HIPEC on the coagulation system, thrombin generation was performed in PPP with 1 pM of TF and quantitatively assessed by ETP (endogenous thrombin potential), LT (lag time), TP (thrombin peak) and TTP (time-to-thrombin-peak) (n = 28, Fig 2). For each patient, data retrieved before chemotherapy, after 30 minutes of chemotherapy, at day 1, day 3 and day 7 postoperative were normalized to preoperative values (Table 3).

**Table 3:** Effect of HIPEC on thrombin generation.

	Before incision	Before chemotherapy		30 min chemotherapy		Day 1		Day 3		Day7	
	median	median	IQR	median	IQR	median	IQR	median	IQR	median	IQR
LT	100	85	72–96	77	69–87	86	77–116	106	84–144	144	125–186
ETP	100	100	84–131	115	100–130	115	86–137	103	84–119	93	70–109
TP	100	111	84–156	127	99–161	148	113–224	121	75–206	113	69–175
TTP	100	90	80–100	83	73–92	80	71–101	97	77–122	121	109–135

Data are presented as percentage of before incision. Significant changes compared to before incision values are underlined. LT lag time; TTP time-to-thrombin peak; ETP endogenous thrombin potential; TP thrombin peak.

During surgery, the LT and TTP reduced (significant shortening after 30 minutes of chemotherapy with p values of 0.0003 and 0.002, respectively) and the TP and ETP increased (significant higher after 30 minutes of chemotherapy with p values of 0.004 and 0.02, respectively). Subsequently, after surgery, the LT and TTP increased and ETP and TP decreased in time (Fig 2). Interestingly, the LT and TTP were prolonged on day 7 compared to before incision (p values of 0.0005 and 0.0007, respectively).

## Discussion

CRS and HIPEC is a therapeutic modality for patients with abdominal malignancies responsible for inducing hemostatic disruption, both on platelet and on coagulation level. To our knowledge, this is the first study demonstrating the combined use of ROTEM, PACT and CAT assays during CRS and HIPEC with a follow-up of 7 days postoperative. This approach demonstrated changes in coagulation much earlier than noticed by standard coagulation tests (PT and aPTT).

*Platelet reactivity* (relative to before incision values) to CRP and TRAP seems to be slightly reduced during CRS and HIPEC with regard to α_IIb_β3 activation, while P-selectin expression was not affected. The decline in α_IIb_β_3_ activation is most likely a consequence of impact of CRS and HIPEC. Furthermore, we observed a strong inter-individual variation in platelet reactivity, as previously demonstrated by others [25]. Certain drugs as used during anesthesia (e.g. aprotinin) also exert a different impact on the granule release potential and the aggregation potential [26].

Moreover, it has been known that in this population, activated leukocytes and endothelial cells express adhesion molecules on their luminal surfaces, and inflammatory mediators (cytokines, chemokines, nitric oxide, and reactive oxygen species) are secreted into the microenvironment, ultimately leading to either a positive platelet response (an enhancement of inflammation) or a negative one (an inhibition of inflammation) [27].

### Thrombin generation measurements demonstrate time dependent changes

Lag time values and time to peak values, resembling the clotting time, decreased early during surgery (30 minutes of chemotherapy) but ended up with significant higher values than before incision, which might be related with the LMWH prophylaxis initiated early in the postoperative phase. The authors acknowledge the limitation imposed by the use of a prophylactic LMWH dose as a potential confounder for the results, requiring further research. Additionally, thrombin peak values progressively increased until day 1 post-operative which could be expected as elevated thrombin generation following surgical trauma is indicative for elevated plasma procoagulants (tissue factor, FVIII) and decreased anti-coagulants [28]. However, thrombin peak values decreased from day 1 to values measured preoperative. The endogenous thrombin potential initially increased after 30 minutes of chemotherapy but decreased in the postoperative period which was not found in trauma patients where measurements indicate that the capacity to generate thrombin was not altered [28].

### Maximum clot firmness decreased during surgery but significantly increased at day 7

ROTEM EXTEM MCF, INTEM MCF and FIBTEM MCF decreased during CRS as mentioned by others [29,30]. These findings are considered a nonspecific consequence of surgical aggression, with MCF FIBTEM deterioration as a consequence of the decrease in the concentration of plasmatic fibrinogen or a deterioration of its function. At day 7, INTEM and FIBTEM MCF values were significantly higher than before surgery.

### No considerable changes in platelet count and hemoglobin concentration and absence of leukopenia

Platelet dynamics were previously described by Perez Ruixo, indicating a model based time course of platelet counts, which simultaneously accounts for the acute-immediate thrombocytopenia response induced by the CRS and the hyperthermic intraperitoneal oxaliplatin effects in bone marrow, as well as the subsequent thrombocytosis due to the natural defence mechanism to prevent major bleedings [31]. Our measurements revealed that during CRS and 30 minutes of chemotherapy, platelet count did not significantly change, however it decreased to non-critical levels on day 1 and 3. Hereafter platelet counts were normalized at day 7. In this context, a correlation between oxaliplatinum exposure and thrombocytopenia has been described with thrombocytopenia related to chemotherapeutic agents, caused by a mild bone marrow suppression (toxicity to megakaryocytic progenitors) occurring in 45–77% of the cases [32, 33]. Additionally, oxaliplatin has also been related with drug-induced, immune mediated thrombocytopenia (DITP) and splenic sequestration of platelets due to portal hypertension based on sinusoidal injury [34–37].

5-FU (Fluorouracil), on the other hand, has been related to infection, bleeding and thromboembolism [38, 39]. Also, 5-FU is known to produce a significant reduction in platelet aggregation and platelet factor-3 (PF(3)) availability without being associated with thrombocytopenia [40]. 5-FU does contribute to a potentially pro-thrombotic environment through the depletion of protein C and increased thrombin activity [41]. Furthermore, animal models and human endothelial cell cultures exposed to 5-FU demonstrated endothelial cell damage with the potential to promote thrombus formation [42]. Another chemotherapeutic agent, cisplatin causes only mild hematological toxicity to all 3 blood lineages [43]. The use of mitomycin however results in a high incidence of bone marrow suppression, particularly thrombocytopenia and leukopenia occurring within 8 weeks after onset of therapy and recovery after cessation within 10 weeks which is beyond the scope of our measurements. [44–46]

With respect to hemoglobin concentrations, significant changes during and after surgery were not noticed in this study. This was partially explained by the minimal blood loss we measured during surgery (123 ± 88 ml) for a mean operation theatre time of 493 (range 293–800) minutes, even when the total amount of peri-operative fluid therapy was 4325 ± 2015 ml and by blood transfusion as was required in 15% of the patients.

The white blood cell count significantly increased early in the process (from incision to the start of chemotherapy). On day 1 and 7 post-operative, the white blood cell count was still significantly higher than before incision which has been reported by others [7–10]. These findings are in contrast with the leukopenia mentioned by others [47]. In our population, only 20% of the patients underwent a splenectomy. Moreover, no relationship was noticed between patients with or without splenectomy on the white blood cell or platelet count variations postoperatively. Preoperative leukopenia related to preoperative chemotherapy is considered an argument to delay the CRS and HIPEC intervention. Although white blood cell counts increased as noticed after any major surgery, immunosuppression counteracted a more significant rise which could be misleading in case of a postoperative infection.

### Potential hemostatic interference in CRS and HIPEC is phase dependent

Disturbance in hemostatic physiology related to extensive surgery and HIPEC can be caused by a combination of factors including: hemodilution, tumor burden effect, consumption lysis, significant protein losses which, alongside with albumin diffusion into extravascular spaces, blood loss, early activation of protein C and FX, direct effect of intra-peritoneal chemotherapy and hyperthermia which all potentially account for the early impact on coagulation. Considerable postoperative fluid volume shifts, hepatic toxicity due to anti-neoplastic agents and direct hepatic trauma potentially account for later effects [7–10]. Volume shifts can result in cellular hypoxia by increasing the tissular diffusion distance for oxygen which might promote a pro-thrombotic state [48].

### Hyperthermia as independent feature influences hemostatic function

Worel et al. and Ludgate demonstrated a temporal correlation between hemostatic alterations and elevation in liver enzymes leading to the assumption that liver impairment might play a crucial role in coagulation disturbances observed during extra-corporeal circulation induced whole body hyperthermia (sarcoma treatment) which is followed by liver sequestration of platelets. [49,50] Bull et al. on the other hand recorded no clotting disorders following whole body hyperthermia [51]. Others concluded that whole body hyperthermia was associated with a consumption coagulopathy [52]. Oglesbee et al. concluded that the induction of whole body hyperthermia by extracorporeal circulation resulted in a coagulopathy characterized by thrombocytopenia, increased plasma fibrin degradation products, prolonged clotting times, and evidence of spontaneous bleeding [53]. Boldt et al. reported that hyperthermic immersion (HI) lead to a shortening of aPTT (P < 0.05) [54]. Fibrinogen concentration decreased immediately after HI (P < 0.05) but increased during recovery (P < 0.05). Plasminogen activator inhibitor (PAI) activity decreased during HI (P < 0.05), D-dimer concentration was not found to change. Platelet count increased (P < 0.05) during HI. The increases in tissue-type plasminogen activator concentration as well as leucocyte count during HI were related to hemoconcentration. PT, PAI-activity and granulocyte count decreased during thermoneutral immersion (P < 0.05). Meyer et al. hypothesized that elevated body temperature and reduced central blood volume contributes to hypercoagulability, possibly related with a moderate sympathetic activation, in critically ill patients [55].

### Hyperglycemia affects thrombin generation

Oxaliplatin is unstable in chloride-containing media, resulting in the use of 5% dextrose as the carrier solution in these procedures. However, the use of chloride-containing carrier solutions for oxaliplatin does not relevantly affect its concentrations under tested in-vitro conditions. Chloride seems to promote formation of the active cytotoxic drug form of oxaliplatin and therefore could enhance its cytotoxic effect [56]. In this study, glucose concentration increased during surgery, peaked during chemotherapy (related to the dextrose 5% solution used for chemotherapy instillation) but continued to be increased on day 3 and 7 postoperative [14]. In diabetic patients, the hyperglycemic byproduct methylglyoxal impairs anticoagulant activity through covalent adduction of antithrombin III [57]. Acute hyperglycemia has been related with a transient increase in thrombin generation [58].

### CRS-HIPEC initially decreases fibrinogen but significantly rises from day 3 postoperative

Fibrinogen decreased initially during surgery but continued to increase postoperatively from day 1 until day 7.

There are at least three mechanisms responsible for the reduction in fibrinogen concentration during major surgery: hemodilution, consumption, and degradation. Volume replacement with crystalloids and colloids, and blood transfusions containing low levels of fibrinogen can result in hemodilution. Second, excessive activation of the hemostatic system, owing to, for example, blood contact with the surface of the HIPEC circuit and operation trauma, occasionally instigate a disseminated intravascular coagulation process with consumption of platelets, fibrinogen, and other coagulation factors. Other mechanisms leading to fibrinogen loss during surgery are fibrinogenolysis or fibrinogen degradation, caused by plasmin-mediated proteolysis after activation of plasminogen through tissue plasminogen activator (t-PA). It is currently unknown to which extent these different mechanisms contribute to the decrease in fibrinogen concentration during CRS/HIPEC [59]. In our study, we observed an increase of ROTEM ML values postoperative which suggests increased fibrinolysis but simultaneously, fibrinogen values already revealed an increasing trend. The increase of fibrinogen in the postoperative period is related to the prominent role for fibrinogen and degradation products in regulating the inflammatory response in several target tissues [60].

The strengths associated with this study is related to the combined ROTEM, PACT and CAT measurements up to 7 days after surgery which is the first time reported in HIPEC literature. Araña et al. limited the measurements only during the surgery [30]. Moreover, the patient populations consisted only of female patients and only ROTEM, but no PACT and CAT assays were used. They concluded that FIBTEM MCF changes were most relevant.

Although this combination of assays covers a broad spectrum of hemostasis testing, the assays are not able to fully quantify and reveal the hemostatic condition in vivo at the site of surgery. Moreover, from the 27 patients enrolled in this study, only 10 patients were tested by the PACT assay. Only patients not treated with acetylsalicylic acid. The inter-individual variations as noticed both in the PACT and CAT assays suggest considerable individual difference in platelet activation and the potential of thrombin generation, which could not be clarified based on the available data. Additionally, adding other tests such as quantifying coagulation factors, tissue factor expression, plasmin generation, etc is considered an opportunity for a follow-up study. Also, TG was only measured in platelet poor plasma whereas a defect in platelet activation requires testing in platelet rich plasma. More research is required to reveal the influence of age, gender and cancer type on the results presented in this paper. Patients scheduled for CRS and HIPEC have considerable variations in oncological histories, therefore we are interested in the interaction of inflammatory functions and the immune response in general on thrombocytes and coagulation in this context. All patients received a prophylactic LMWH dose the evening before surgery which could have altered our findings (prolongation of the LT and reduction of ETP).

This study does however provide an extra argument to widen the spectrum of tests used to quantify different aspects of hemostasis and interfere where necessary (e.g. administration of LMWH based on LT and TTP values) although the authors acknowledge the small sample size as a limitation of the study.

## Conclusion

Based on this study, the impact of CRS-HIPEC on high precision coagulation measurements (TG and high precision platelet function measurements (PACT)) is quantitatively illustrated from the moment of incision up to 7 days after surgery. This study demonstrated that PT, aPTT and platelet count insufficiently demonstrated the impact of surgical stress, hyperthermia, chemotherapy and considerable fluid shifts on the overall hemostatic physiology of CRS and HIPEC. This study suggests a decreased platelet reactivity and an altered thrombin generation pattern.

Longer follow-up in a larger patient cohort will be required to clarify the inter-individual variations of postoperative bleeding or thrombosis (DVT/PE) in patients treated with CRS and HIPEC.

## Supporting information

S1 File STROBE_checklist_cohort PONE-D-17-01312R1. (DOCX)

S2 File Data1. (XLSX)

S3 File TGHIPACAT. (XLSX)

S4 File ROTEM. (XLSX)

S5 File PlateletFunction. (XLSX)
